# Risk assessment and source analysis of heavy metals in soil around an asbestos mine in an arid plateau region, China

**DOI:** 10.1038/s41598-024-58117-4

**Published:** 2024-03-30

**Authors:** Xuwei Li, Da Ding, Wenyi Xie, Ya Zhang, Lingya Kong, Ming Li, Mei Li, Shaopo Deng

**Affiliations:** 1 Ministry of Ecology and Environment of China, Nanjing Institute of Environmental Sciences, Nanjing, 210042 China; 2 State Environmental Protection Key Laboratory of Soil Environmental Management and Pollution Control, Nanjing, 210042 China

**Keywords:** Environmental sciences, Environmental impact

## Abstract

Asbestos is widely used in construction, manufacturing, and other common industrial fields. Human activities such as mining, processing, and transportation can release heavy metals from asbestos into the surrounding soil environment, posing a health hazard to the mining area's environment and its surrounding residents. The purpose of the present study was to determine the extent of ecological and human health damage caused by asbestos pollution, as well as the primary contributors to the contamination, by examining a large asbestos mine and the surrounding soil in China. The level of heavy metal pollution in soil and sources were analyzed using methods such as the geo-accumulation index (I_geo_), potential ecological risk index (RI), and positive matrix factorization (PMF) model. A Monte Carlo simulation-based health risk model was employed to assess the health risks of heavy metals in the study area’s soil to human beings. The results showed that the concentrations of As, Pb, Cr, Cu, and Ni in the soil were 1.74, 0.13, 13.31, 0.33, and 33.37 times higher than the local soil background values, respectively. The I_geo_ assessment indicated significant accumulation effects for Ni, Cr, and As. The RI evaluation revealed extremely high comprehensive ecological risks (RI ≥ 444) in the vicinity of the waste residue heap and beneficiation area, with Ni exhibiting strong individual potential ecological risk (Eir ≥ 320). The soil health risk assessment demonstrated that As and Cr posed carcinogenic risks to adults, with mean carcinogenic indices (CR) of 1.56E − 05 and 4.14E − 06, respectively. As, Cr, and Cd posed carcinogenic risks to children, with mean CRs of 1.08E − 04, 1.61E − 05, and 2.68E − 06, respectively. Cr also posed certain non-carcinogenic risks to both adults and children. The PMF model identified asbestos contamination as the primary source of heavy metals in the soil surrounding the asbestos mining area, contributing to 79.0%. According to this study, it is recommended that management exercise oversight and regulation over the concentrations of Ni, Cr, Cd, and As in the soil adjacent to asbestos mines, establish a designated control zone to restrict population activities, and locate residential zones at a safe distance from the asbestos mine production zone.

## Introduction

In recent years, with the rapid development of the national industry, soil environmental issues have become increasingly prominent and have attracted widespread attention from society. As one of the most common soil pollutants, heavy metals possess characteristics such as persistence, accumulation, and difficulty in degradation^1^. When pollution reaches a certain level, it can make the natural ecological environment fragile and directly or indirectly threaten human health and the sustainable development of society^2^. Mineral resource development has always been one of the main sources of heavy metal pollution in environmental media^3^. Currently, many scholars have conducted research on heavy metal pollution in different types and regions of mines and surrounding soils. The results show that heavy metals generated by mining activities have caused varying degrees of pollution to surrounding soils. For example, Zhang et al. discovered that the soils in 15 lead–zinc mining regions in southern China were polluted with a diverse range of heavy metals, predominantly Cd, Pb, and Zn^4^. Sun et al. found that small-scale mining activities resulted in average concentrations of Cu, Zn, As, Cd, and Pb in surface soils of farmland being seven times higher than the corresponding background values^5^. Mohammad et al. assessed the pollution level of farmland surrounding a coal mine in Bangladesh and found significant accumulations of Mn, Zn, and Pb, primarily derived from coal mining activities^6^. Vuong et al. conducted a study in Vietnam where they gathered 17 surface soil samples from a lead–zinc mining region. Their findings revealed that Pb, Zn, and Cd were the most heavily contaminated substances^7^. Hye-Sook Lim et al. conducted a comprehensive assessment of soil heavy metal pollution near an abandoned gold mine in South Korea and found that the concentrations of As and Hg in farmland soil reached as high as 626 mg·kg^−1^ and 4.9 mg·kg^−1^, respectively. The highest hazard quotient and carcinogenic risk for the mine were 16 and 2.7E-03, respectively^8^.

Asbestos is known for its high tensile strength, resistance to chemical degradation, and excellent heat resistance. These characteristics have led to its widespread use in various industries such as construction and manufacturing^9^. Long-term asbestos mining generates a large number of tailings. For example, there are still over 2800 operational mines in India^10^. Additionally, countries like Greece^11^, Canada^12^, Russia^13^, and Italy^14^ also have asbestos mines in operation or in a state of closure. In China, asbestos mining production in 2020 has reached a total of 100,000 t^15^. Industrially, for every ton of asbestos consumed, approximately 10 g of asbestos fibers are released into the environment, the released short asbestos fibers can remain suspended in the atmosphere for several months^16^. Fine asbestos fibers that settle on the surface of soil can be transported by wind, even floating several km away, causing pollution to mining areas and surrounding environments^17^. In recent years, research on asbestos in mining areas and surrounding soil has attracted increasing attention from researchers. Especially, considering the toxicity of asbestos to health, some public health institutions primarily study the length of asbestos fibers and their potential pathogenicity upon inhalation by humans^9,18,19^. During the process of pollution dispersion, asbestos is often accompanied by heavy metal elements such as Ni, Cd, Cr^20^, which can spread to the site and surrounding soil, posing a serious threat to human health and ecological environment.

However, currently, there is still insufficient research on the asbestos mines and heavy metal contamination in the surrounding soil, both domestically and internationally. Especially in terms of human health risks and source analysis, only Sonali Banerjee et al. have evaluated the soil pollution and health risks in agricultural fields near an asbestos mine in India using synergistic statistical methods. The results indicated higher ecological risks of heavy metals near the mine, as well as carcinogenic risks for children and adults^20^. Adarsh Kumar et al. found that the concentrations of Ni and Cr in the soil of agricultural fields near a Cr-containing asbestos mine in India exceeded the soil threshold values^21^. The aforementioned research primarily examined the effects of asbestos mines on adjacent agricultural land in India. China, as a major asbestos mining country, has more sensitive receptors in and around asbestos mines, but the sources of asbestos contamination, ecological risks, and health risks have rarely been reported. In addition, traditional health risk assessment uses fixed values as input parameters, which may overestimate or underestimate the risk, whereas probabilistic risk assessment uses parameters with probability distributions as input values, which is able to deal with the uncertainty and variability of data parameters.

This study examines the production and residential areas of asbestos mines in China. It evaluates the pollution characteristics and potential ecological risks of heavy metals using various methods such as the geo-accumulation index method, potential ecological risk index method, positive matrix factorization method, and probabilistic health risk assessment. The study also analyses the sources of heavy metal pollution in asbestos mine sites and assesses the health risks to nearby sensitive receptors. The aim is to provide technical support for the precise prevention and control of heavy metal pollution in asbestos mine environments.

This article analyzes the content characteristics of heavy metals in an asbestos mine and its surrounding soils in an arid plateau region. It assesses the potential ecological risks, environmental risk levels, and scope of heavy metal pollution in the asbestos mining area. Additionally, it utilizes the Positive Matrix Factorization (PMF) method and Monte Carlo simulation to conduct source analysis and probabilistic assessment of health risks associated with heavy metals in the soil. The goal is to provide technical support for precise prevention or control of heavy metal pollution in asbestos mining environments.

## Materials and methods

### Study area overview

The study area includes the asbestos mining area and the residential area located on the northeast side. The mining area has a length of approximately 6 km from east to west, a width of approximately 4 km from north to south, and covers an area of about 14.11 km^2^. The mining area has a history of 62 years and is a large ultrabasic rock-type chrysotile deposit. The main mineral of asbestos ore in the mining area is clinochrysotile and the mining method used is spiral open-pit mining. Asbestos fibers in the soil of the mining area mainly come from natural weathering and erosion of asbestos mines, as well as human mining activities and the deposition of tailings and waste residues. The waste residues are piled around the mining area and distributed to the south of the mining area. The size of the deposition areas varies, and their heights typically range from 3 to 8 m, with the maximum height not exceeding 15 m. The study area has a continental plateau climate, characterized by dry and cold conditions with drastic temperature changes. It is windy throughout the year, and according to meteorological data, the average annual temperature in the mining area is 1.5 °C. The area experiences minimal rainfall, with an annual precipitation of only 46.9 mm. The daily average wind speed is generally greater than 5 m·s^−1^, with a maximum of 26 m·s^−1^. Hydrogeological data and geological exploration results of surrounding mines indicate that no groundwater has been observed within a depth of 200 m in the currently exploited mines.

### Sample collection and analysis methods

**Figure 1:**
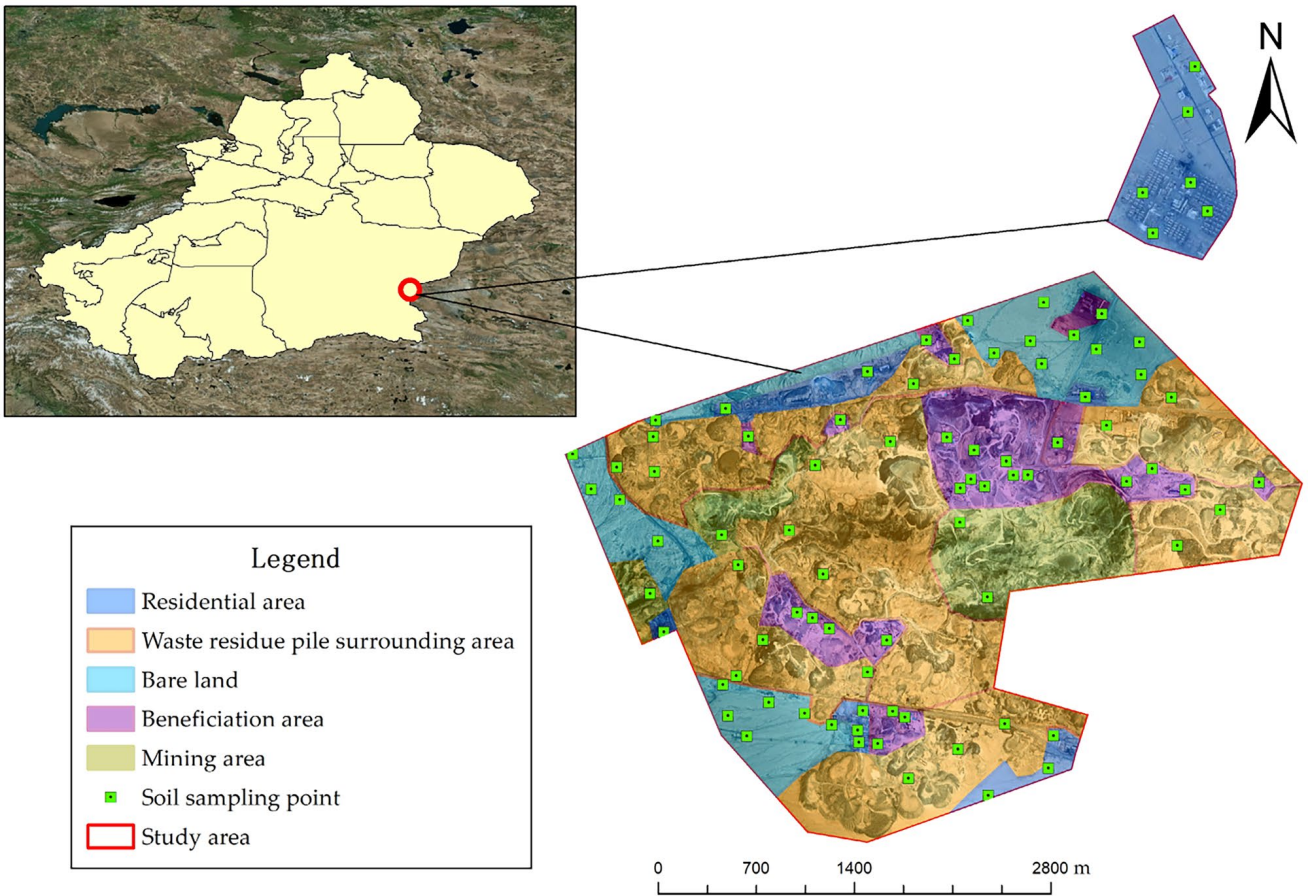
Distribution and zoning map of sampling points in the study area. Map was created using ArcGIS Desktop 10.3 (https://www.esri.com/en-us/arcgis/products/arcgis-pro/overview).

In this study, a total of 84 sampling points were set up in the investigation area, distributed in areas such as the mining area, beneficiation area, waste residue pile surrounding area, residential area, and bare land. After scraping off impurities with a wooden shovel, the surface 0–30 cm of soil was collected. Each sample weighed approximately 1 kg, and a total of 84 samples were collected. Considering that the main sources of soil asbestos contamination are asbestos ore mining, as well as asbestos tailings, asbestos products, and asbestos dust generated during asbestos processing, four types of pollution source samples were collected for heavy metal concentration analysis, with three samples collected for each pollution source. The location map of the study area is shown in Fig. 1.

For the analysis methods and detection limits of Cr, the method described in “Soil Quality—Determination of Total Chromium—Flame Atomic Absorption Spectrophotometry” (HJ 491–2019) was referenced. For other elements, the analysis methods and detection limits were in accordance with the requirements specified in “Soil Environmental Quality—Risk Control Standard for Soil Contamination of Development Land (Trial)” (GB 36,600–2018), specifically in “Table 3: Analysis Methods for Soil Pollutants”. The testing methods for the samples included graphite furnace atomic absorption spectrophotometry, atomic fluorescence spectrometry, flame atomic absorption spectrophotometry, etc.

### Geo-accumulation index method

The Geo-accumulation Index (I_geo_) is a quantitative indicator proposed by German scientist Müller in the 1970s to study the degree of heavy metal pollution in sediments and other substances^22^. What sets the Geo-accumulation Index method apart from other pollution assessment methods is that it takes into account the factors that cause background value changes due to natural diagenesis. The detailed calculation process is formulated in the following Eq. (1).

1 $${{\text{I}}}_{{\text{geo}}}={{\text{log}}}_{2}[{{\text{C}}}_{{\text{i}}}/({\text{K}}\cdot {{\text{C}}}_{0})]$$

In the formula, I_geo_ represents the geo-accumulation index of heavy metal i; Ci represents the measured value of heavy metal i in soil (mg·kg^−1^); C_0_ represents the background value of element i in soil (mg·kg^−1^); k is the correction coefficient for the variation of background values caused by soil-rock differences, which is generally taken as 1.5. Taking into account the regional characteristics of the study area, the composition of parent materials in surface soils, and the evolutionary laws of geomorphology and geological environment, this study takes the soil element background values of non-agricultural land in Ruoqiang County, Xinjiang as a reference. The values for Hg, Cd, As, Pb, Cu, Ni, Cr, and Zn are 0.02, 0.12, 11.20, 19.40, 26.27, 26.60, and 68.80 mg·kg^−1^, respectively^23^.

The classification criteria for geo-accumulation index are as follows: Igeo ≤ 0 indicates no pollution; 0 < I_geo_ ≤ 1 indicates slight pollution; 1 < I_geo_ ≤ 2 indicates moderate pollution; 2 < I_geo_ ≤ 3 indicates severe pollution; I_geo_ > 3 indicates extremely severe pollution. When the I_geo_ value is greater than 0, it indicates that the main source of heavy metals in the soil is human activities rather than natural factors such as parent materials.

### Potential ecological risk index method

The potential ecological risk index method was proposed by Swedish scientist Hakanson. It takes into account the properties of heavy metals, their environmental behavior, toxic effects, synergistic effects between different heavy metals, and the sensitivity of the ecological environment to heavy metals^24^. The formula for calculating the individual potential ecological risk index (${{\text{E}}}_{{\text{r}}}^{{\text{i}}}$) is as following Eq. (2).

2 $${{\text{E}}}_{{\text{r}}}^{{\text{i}}}=\frac{{{\text{T}}}_{{\text{r}}}^{{\text{i}}}\cdot {{\text{C}}}_{{\text{i}}}}{{{\text{C}}}_{0}}$$

In the formula, ${{\text{T}}}_{{\text{r}}}^{{\text{i}}}$ represents the toxicity response coefficient of a certain heavy metal in the soil (the toxicity response coefficients for Hg, Cd, As, Pb, Cu, Ni, Cr, and Zn are 40, 30, 10, 5, 5, 5, 2, and 1, respectively). Ci represents the concentration of heavy metals in the soil, and C_0_ represents the background value of heavy metals in the soil. The comprehensive Potential Ecological Risk Index (RI) is the sum of individual potential ecological risk indices, and the calculation formula is as following Eq. (3).

3 $${\text{RI}}=\sum_{{\text{i}}=1}^{{\text{n}}}{{\text{E}}}_{{\text{r}}}^{{\text{i}}}$$

The grading criteria for the Potential Ecological Risk Index (RI) takes into account the toxicity coefficients of eight heavy metal pollutants and the comprehensive evaluation of ecological risk levels^25^. Hakanson proposed the RI grading criteria based on the sum of the toxicity coefficients of As, Cd, Cr, Cu, Hg, Ni, Pb, Zn, and polychlorinated biphenyls (PCBs), which totals to 133. The threshold value for the first level of RI classification according to Hakanson is 150. However, this study involves eight different heavy metals: As, Cd, Cr, Cu, Hg, Ni, Pb, and Zn, and the sum of the toxicity response coefficients for these eight heavy metals in the RI grading criteria is 98. Therefore, the RI classification limits need to be adjusted. To make the adjustment, the study proposes calculating the unit toxicity coefficient grading value as RI = 150/133 = 1.13. The corresponding threshold value for the first level of RI is approximately 111. Each subsequent level’s threshold value is twice that of the previous level^26^. For the adjusted RI grading criteria, please refer to Table 1.

**Table 1:** Grading criteria for potential ecological risk coefficient (${{\text{E}}}_{{\text{r}}}^{{\text{i}}}$) and potential ecological risk index (RI).

Level	${{\text{E}}}_{{\text{r}}}^{{\text{i}}}$	Ecological risk level	RI	Ecological risk level
1	${{\text{E}}}_{{\text{r}}}^{{\text{i}}}$< 40	Slight	RI < 111	Low
2	40 ≤ ${{\text{E}}}_{{\text{r}}}^{{\text{i}}}$ < 80	Moderate	111 ≤ RI < 222	Moderate
3	80 ≤ ${{\text{E}}}_{{\text{r}}}^{{\text{i}}}$ < 160	Strong	222 ≤ RI < 444	High
4	160 ≤ ${{\text{E}}}_{{\text{r}}}^{{\text{i}}}$ < 320	Very strong	RI ≥ 444	Extremely high
5	${{\text{E}}}_{{\text{r}}}^{{\text{i}}}$≥ 320	Extremely strong		

### Monte Carlo simulation for health risk assessment

An evaluation of carcinogenic and non-carcinogenic risks for adults and children through three exposure pathways (oral ingestion, dermal contact, and inhalation) was conducted using the health risk assessment model recommended by the United States Environmental Protection Agency (USEPA). The daily soil intake for oral ingestion, dermal contact, and inhalation can be calculated according to Eqs. (4), (6).

4 $${{\text{ADD}}}_{{\text{ingest}}}={{\text{C}}}_{{\text{i}}}\cdot {{\text{R}}}_{{\text{ingest}}}\cdot {\text{EF}}\cdot {\text{ED}}/{\text{BW}}\cdot {\text{AT}}\cdot {10}^{-6}$$

5 $${{\text{ADD}}}_{{\text{dermal}}}={{\text{C}}}_{{\text{i}}}\cdot {\text{SA}}\cdot {\text{SL}}\cdot {\text{ABF}}\cdot {\text{EF}}\cdot {\text{ED}}/{\text{BW}}\cdot {\text{AT}}\cdot {10}^{-6}$$

6 $${{\text{ADD}}}_{{\text{inhal}}}={{\text{C}}}_{{\text{i}}}\cdot {{\text{R}}}_{{\text{inhal}}}\cdot {\text{EF}}\cdot {\text{ED}}/{\text{PEF}}\cdot {\text{BW}}\cdot {\text{AT}}$$

In the formulas, ADD_ingest_, ADD_dermal_, and ADD_inhal_ represent the daily average intake of heavy metals from soil through ingestion, dermal contact, and inhalation, respectively. Ci represents the measured value of heavy metal i in soil. The meanings of other parameters can be found in Table 2.

**Table 2:** Input parameters and values in health risk assessment for soil with Mote Carlo.

Parameter	Description	Unit	Type	Children	Adult	Reference
R_ingest_	Soil ingestion rate	mg·d^−1^	Triangular	66, 103, 161	4, 30, 52	^27^
EF	Exposure frequency	d·y^−1^	Point	350	350	^28^
ED	Exposure duration	y	Uniform	0,10	0,50	^29^
R_inhal_	Inhalation rate	m^3^·d^−1^	Point	7.6	20	^30^
PEF	Particle emission factor	m^3^·kg^−1^	Point	1.36E + 09	1.36E + 09	^29^
BW	Average body weight	kg	Lognormal	16.68, 1.48	57.03, 1.18	^31^
AT	Average time	d	Point	2190	9125	^31^
SA	Exposed skin area	cm^2^	Lognormal	7422, 1.25	18,182, 1.1	^31^
AF	Skin adherence factor	mg·cm^−2^	Lognormal	0.65, 1.2	0.49, 0.54	^32^
ABF	Dermal adsorption factor	–	Point	0.001	0.001	^29^

–data not available.

Calculate the non-carcinogenic risk index and carcinogenic risk index through the daily average soil intake, which are Eqs. (7), (8).

7 $${\text{HI}}=\sum {\text{HQ}}=\sum ({{\text{ADD}}}_{{\text{ij}}}/{{\text{RfD}}}_{{\text{ij}}})$$

8 $${\text{TCR}}=\sum {\text{CR}}=\sum {{\text{ADD}}}_{{\text{ij}}}\cdot {{\text{SF}}}_{{\text{ij}}}$$

In the formulas, HQ and HI represent single and integrated non-carcinogenic risk indices, respectively. CR and TCR represent single and integrated carcinogenic risk indices, respectively. RfD_ij_ refers to the reference dose, while SF_ij_ represents the slope factor, with specific values given in Table 3. When HQ/HI ≤ 1, it indicates that the non-carcinogenic risk can be ignored. Conversely, there is a non-carcinogenic risk. When CR/TCR ≤ 1.00E − 6, it suggests that the carcinogenic risk can be ignored. If 1.00E − 6 < CR/TCR ≤ 1.00E − 4, it indicates the presence of tolerable carcinogenic risk. Finally, if CR/TCR > 1.00E − 6, it implies the existence of intolerable carcinogenic risk.

**Table 3:** Corresponding reference dose (*RfD*) and slope factors (*SF*) values of heavy metals in soils in health risk assessment model with Mote Carlo simulator.

Elements	RfD (mg·(kg day)^−1^)			SF ((kg day) mg^−1^)			References
	Ingestion	Inhalation	Dermal	Ingestion	Inhalation	Dermal	
As	3.00E − 04	1.23E − 04	1.23E − 04	1.5E + 00	1.51E + 00	3.66E + 00	^33^
Cd	1.00E − 03	1.00E − 05	1.00E − 05	6.1E + 00	6.30E + 00	–	^33^
Cu	4.00E − 02	4.02E − 02	1.20E − 02	–	–	–	^34^
Cr	3.00E − 03	2.86E − 05	6.00E − 05	8.50E − 03	4.20E + 00	–	^33^
Hg	3.00E − 04	8.57E − 05	2.10E − 05	–	–	–	^32^
Ni	2.00E − 02	2.06E − 02	5.40E − 03	–	8.4E − 01	–	^33^
Pb	3.50E − 03	3.52E − 03	5.25E − 04	8.50E − 03^e^	–	–	^34^
Zn	3.00E − 01	3.00E − 01	6.00E − 02	–	–	–	^34^

- data not available.

Compared to traditional health risk models, the Monte Carlo simulation-based health risk assessment model first needs to determine the distribution function of variables. Then, random sampling is performed from the variable distribution, and the probability distribution of the simulation results is output^35^. In this study, Oracle Crystal Ball software was used for data processing, with the number of iterations set to 10,000 for each run and a confidence level of 95% to obtain an approximate solution for the risk assessment.

### PMF model analysis method

The PMF (Positive Matrix Factorization) model is a novel and effective source analysis method^36^. By combining the markers of various emission sources and computational results, it infers the types of pollution sources and their contributions to soil heavy metals. The analysis results are more in line with the actual situation. In recent years, this model has been increasingly applied in the identification and allocation of soil pollution sources^37,38^. PMF is a multivariate receptor model that decomposes an i × j-dimensional matrix (x_ij_) into a contribution matrix (g_ik_) and a factor matrix (f_kj_), the calculation formula is as following Eq. (9).

9 $${{\text{x}}}_{{\text{ij}}}=\sum_{{\text{k}}=1}^{{\text{p}}}{{\text{g}}}_{{\text{ik}}}{{\text{f}}}_{{\text{kj}}}+{{\text{e}}}_{{\text{ij}}}$$

In the formula, i and j represent the number of samples and species, respectively, while k represents the number of factors, and e_ij_ represents the residual fraction. The PMF model obtains factor distribution maps and contributions by minimizing the objective function Q using the least square method, the calculation formula is as following Eq. (10).

10 $${\text{Q}}=\sum_{{\text{i}}=1}^{{\text{n}}}\sum_{{\text{j}}=1}^{{\text{m}}}\left(\frac{{{\text{e}}}_{{\text{ij}}}}{{{\text{U}}}_{{\text{ij}}}}\right).$$

Here, U_ij_ represents the uncertainty of each species detected in each sample.

In this study, the following processing steps were performed on the obtained heavy metal concentrations to ensure the accuracy of the research: (1) For non-detects, the concentration was replaced with 5/6 of the detection limit. (2) The number of factors was set to 3–6, and multiple model reconstructions were performed to stabilize the Q value. The ratio residuals and R^2^values were adjusted to ensure that the proportion of component ratio residuals between + 3 and − 3 was greater than 95.5% and that the R^2^ value exceeded 0.98.

### Experimental data processing

The statistical analysis of data for the eight heavy metals was carried out using Excel spreadsheets. In this study, ArcGIS 10.2 geographic information system software was used for inverse distance weighting (IDW) interpolation. The data processing for health risk assessment model based on Monte Carlo simulation was performed using Oracle Crystal Ball 11.1.2.4 software. The source analysis of soil heavy metals was conducted using PMF 5.0.

## Results and discussion

### Characteristics of heavy metal contents in soil

The concentration characteristics of heavy metals in soil are usually described using representative statistical parameters. The statistical characteristic values of heavy metal content in the study area soil are shown in Table 4. It can be seen that, except for Hg, Cd, and Zn, the average concentrations of the other five heavy metals are higher than the soil background values of non-agricultural land in Ruoqiang County to varying degrees. The average concentrations of As, Pb, Cr, Cu, and Ni are 1.74, 0.13, 13.31, 0.33, and 33.37 times higher than the soil background values, respectively. The proportions of samples exceeding the soil background values for seven elements including As, Cd, Pb, Cr, Cu, Zn, and Ni are 57.14%, 36.90%, 63.10%, 91.67%, 70.24%, 23.81%, and 98.81%, respectively. The above results indicate that, in non-agricultural land in Ruoqiang County, except for the soil background value of Hg, which is significantly higher than that of Hg in Xinjiang Autonomous Region, the background values of other elements are lower than the soil background values for those elements in the Xinjiang Autonomous Region^39^. In terms of the proportion of samples exceeding the Ruoqiang County background values, As, Zn, and Ni are all above 50%, indicating that these three elements are widely enriched in the soil. The multiples of Cr and Ni average concentrations exceeding the background values are much larger than those of other elements. Research by Alloway et al. showed that the average concentration of Cr in soils containing serpentinite reaches as high as 3000 mg·kg^−1^, while the concentration range of Cr in uncontaminated soils is 0.5 ~ 250 mg·kg^−1^^40^. Reeves et al. found that the Cr content in serpentinite soil environments in Costa Rica ranged from 1400 to 3640 mg·kg^−1^^41^. Ni and Cr were found to be associated and present in all types of rocks, with high concentrations of Ni primarily observed in serpentine soil^42^. In a serpentine mining waste site in Taiwan Region, the content of Ni ranged from 691 to 1220 mg·kg^−1^^43^. Similarly, near an abandoned asbestos mine in India, the concentration of Ni in the soil was also relatively high, ranging from 945 to 1620 mg·kg^−1^^21^. These findings reveal that the concentrations of Ni and Cr are generally high in serpentine rock formations and the associated soils, consistent with the asbestos mine type and the levels of the two heavy metal pollution observed in this study. This further confirms the severe contamination of surrounding soils caused by this type of mineral deposit.

**Table 4:** Statistical information of heavy metal contents in soil; unit: mg·kg^−1^.

Statistics	As	Hg	Cd	Pb	Cr	Cu	Zn	Ni
Minimum value	1.29	0.002	0.04	2.72	24.91	4.00	16.58	15.25
Maximum value	206.10	0.103	0.29	175.38	1826.35	117.74	122.20	1951.19
Average value	21.79	0.010	0.09	16.62	580.04	20.25	41.72	636.93
Standard deviation	31.54	0.011	0.04	18.30	559.74	13.82	15.95	632.68
Coefficient of variation	1.45	1.18	0.38	1.10	0.97	0.68	0.38	0.99
Background value*	7.94	11.48	0.10	14.67	40.54	15.23	48.00	18.53

The background value* refers to the background value of each element in the non-agricultural land soil of Ruoqiang County, Xinjiang^23^.

The coefficient of variation is a parameter that represents the uniformity of element distribution in soil. A higher coefficient of variation indicates a more uneven distribution and greater disturbance from human activities^44^. The coefficient of variation ranges from 0.38 to 1.45 for eight heavy metals, with the order of magnitude being As > Hg > Pb > Ni > Cr > Cu > Cd > Zn. The coefficients of variation for As, Hg, Pb, Ni, Cr, are greater than 0.9, indicating significant spatial distribution variations and strong heterogeneity. On the other hand, the coefficients of variation for Cd and Zn range from 0.3 to 0.6, indicating moderate variation and relatively consistent influence from external factors.

From Table 5, it can be seen that the average concentrations of As, Hg, Cd, Pb, Cr, Cu, Zn, and Ni in the four types of asbestos pollution sources (asbestos ore, asbestos tailings, asbestos products, and asbestos dust) are 14.73, 0.02, 0.07, 6.23, 1339.17, 10.33, 13.33, and 1788.33 mg·kg^−1^ respectively. Among them, the average concentrations of As, Ni, and Cr exceed the soil background values in the non-agricultural land of Ruoqiang County by 0.9, 95.5, and 32.0 times respectively. Research conducted by Evangelos Gidarakos and others has shown that, except for the high concentrations of Ni and Cr, the concentrations of other heavy metals and harmful substances in the soil are at low levels^45^, which is consistent with the findings of this study. The coefficient of variation for the eight heavy metals ranges from 0.08 to 0.47, while the variation coefficients for Pb and Cu range from 0.3 to 0.6, indicating moderate variation. The variation coefficients for the remaining heavy metals range from 0.1 to 0.3, indicating low variation. This suggests that there is no significant spatial difference in the concentrations of the six elements, including As, Hg, Cd, Cr, Zn, and Ni, with higher levels observed for Ni and Cr.

**Table 5:** Statistical information of heavy metal contents in asbestos pollution sources.

Statistics	As	Hg	Cd	Pb	Cr	Cu	Zn	Ni
Minimum value	8.87	0.016	0.038	3.39	890	4	13	1600
Maximum value	17.1	0.022	0.094	13.2	1560	16	20	2000
Average value	14.73	0.02	0.07	6.23	1339.17	10.33	13.33	1788.33
Standard deviation	2.10	0.00	0.02	2.93	218.50	3.50	2.29	135.70
Coefficient of variation	0.14	0.10	0.27	0.47	0.16	0.34	0.17	0.08

### Soil heavy metal pollution assessment

**Figure 2:**
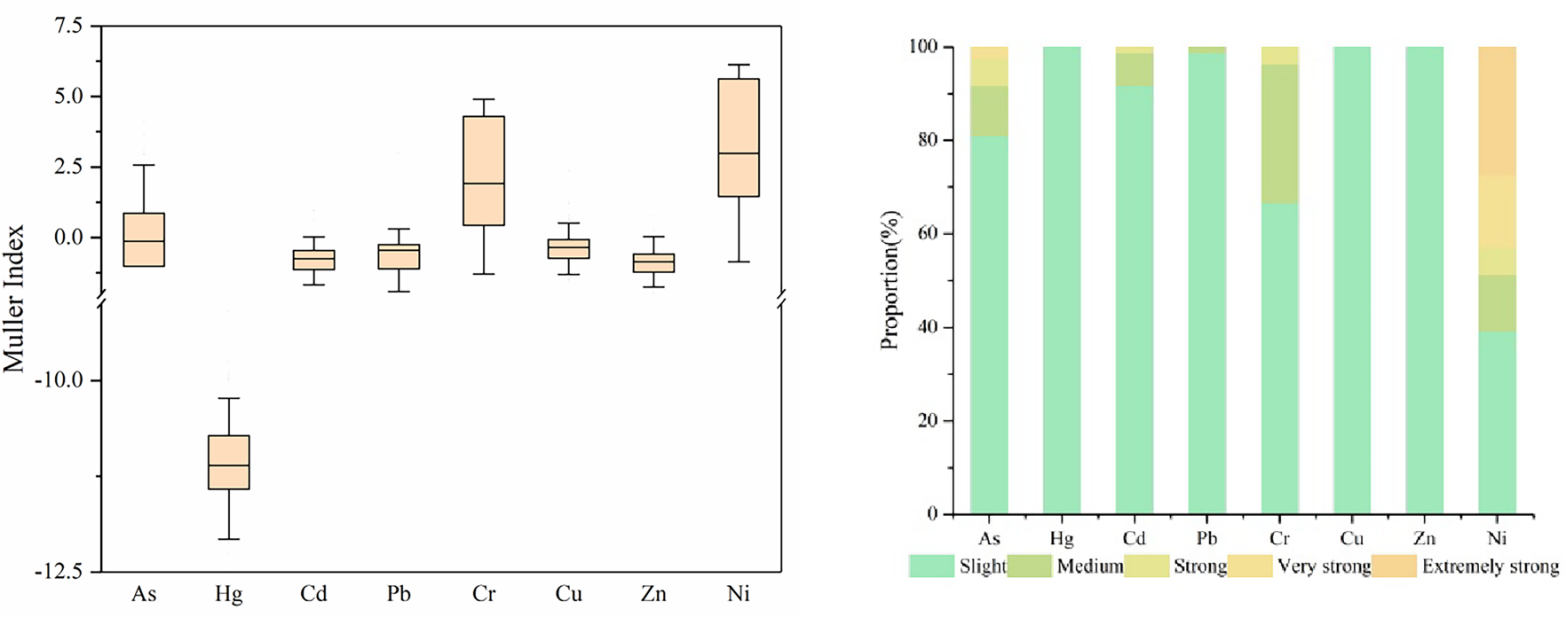
Geoaccumulation index of soil heavy metals in the study area.

The geo-accumulation index method takes into account both geological background and human activities. The mining activities of asbestos mines can to some extent cause soil heavy metal pollution. The evaluation results of the geo-accumulation index for soil heavy metals are shown in Fig. 2. The average values of the geo-accumulation index (I_geo_) are as follows: Ni (3.58) > Cr (2.46) > As (0.04) > Cu (− 0.36) > Pb (− 0.71) > Cd (− 0.74) > Zn (− 0.85) > Hg (− 11.09). Among them, Ni, Cr, and As have evaluation average values greater than 0, indicating the presence of severely polluted sampling sites. The evaluation result for Ni is mainly severe pollution (I_geo_ > 3), accounting for 50%, followed by heavy pollution (2 < I_geo_ ≤ 3) at 17.86%, and only 5.95% of the sampling sites show no pollution (I_geo_ ≤ 0). For Cr, the evaluation result is mainly severe pollution (I_geo_ > 3), accounting for 46.43%, followed by moderate pollution (1 < I_geo_ ≤ 2) at 19.05%, and 16.67% of the sampling sites show no pollution (I_geo_ ≤ 0). The evaluation result for As is mainly no pollution (I_geo_ ≤ 0), accounting for 51.19%, followed by slight pollution (0 < I_geo_ ≤ 1) at 25%, and the percentage of severely polluted sites (I_geo_ > 3) is 3.57%. The evaluation results for Cu, Pb, Cd, and Zn are mainly no pollution (0 ≤ I_geo_ < 1), accounting for 78.57% to 100% of the sampling sites. The evaluation result for Hg is no pollution (I_geo_ < 0), accounting for 100% of the sampling sites.

The above results indicate that the cumulative effects of Ni, Cr, and As elements in the study area are significant under the influence of asbestos production and human activities. Among them, the mean ranks of the geo-accumulation index for Ni and Cr are the highest, indicating severe pollution. Kahangwa et al. demonstrated that the ground accumulation index is indicative of soil contamination levels, with Cr being the primary contaminant in the research area^46^. Adarsh Kumar et al.’s research shows that the geo-accumulation index of Ni and Cr near abandoned asbestos mines in agricultural soil is relatively high, with maximum values of 4.37 and 3.59, respectively. These values decrease with increasing soil depth^21^. This finding is consistent with the conclusions of this study. As for As, 3.57% of the sampled points show a state of severe enrichment, indicating that local sites are more significantly affected by human activities. This result is also consistent with the conclusion reflected by the variation coefficient of As element. Jiang et al.’s research on nonferrous metal mines in northwestern China also indicates that As is greatly influenced by human activities, with a geo-accumulation index ranging from − 0.838 to 7.34 and a proportion of severely polluted sites (I_geo_ > 5) of 21.4%^47^. Hence, potential pollution with additional heavy metals resulting from human activities during the mining and manufacture of asbestos.

### Potential ecological risk assessment

**Figure 3:**
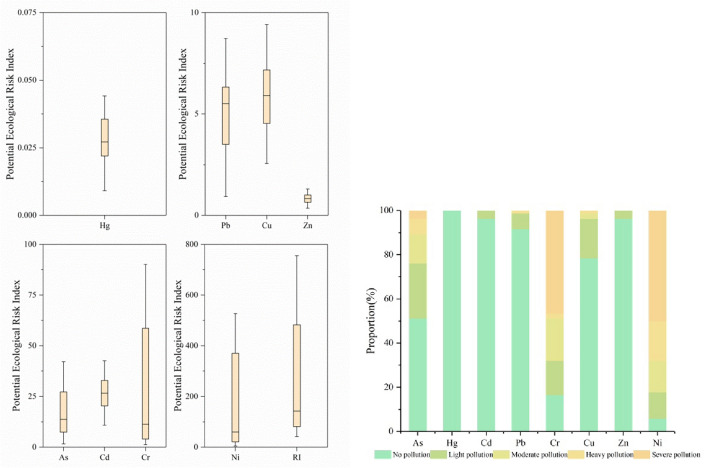
Individual potential ecological risk index of soil heavy metals and proportion of sampling points.

Potential ecological risk is generally influenced by factors such as the properties of heavy metals, their biological toxicity, and ecological effects. The results of the potential ecological risk index assessment in the study area are shown in Fig. 3. According to the findings of the ecological risk assessment conducted on the sampling sites, the average values of individual potential ecological risks (${{\text{E}}}_{{\text{r}}}^{{\text{i}}}$) are as follows: Ni (171.87) > Cr (28.62) > Cd (28.25) > As (27.45) > Cu (6.65) > Pb (5.67) > Zn (0.87) > Hg (0.03). Ni, Cr, Cd, As, and Pb pose moderate or higher ecological risks. Among them, the proportion of sampling points evaluated as having extremely high ecological risk (${{\text{E}}}_{{\text{r}}}^{{\text{i}}}$ ≥ 320) for Ni is 27.38% of the total sampling points. The proportions of sampling points evaluated as having very high ecological risk (160 ≤ ${{\text{E}}}_{{\text{r}}}^{{\text{i}}}$  < 320) for As and Ni are 15.48% and 2.38%, respectively. The proportions of sampling points evaluated as having high ecological risk (80 ≤ ${{\text{E}}}_{{\text{r}}}^{{\text{i}}}$  < 160) for As, Cd, Cr, and Ni are 5.95%, 1.19%, 3.57%, and 5.95%, respectively. The proportion of sampling points evaluated as having high ecological risk or above for Ni reaches 48.81%. Overall, the evaluation results for the eight heavy metals mainly indicate slight ecological risks (${{\text{E}}}_{{\text{r}}}^{{\text{i}}}$ < 40), with the proportion of sampling points falling within the range of 39.29% to 100%. The proportion of sampling points evaluated as having slight ecological risk (${{\text{E}}}_{{\text{r}}}^{{\text{i}}}$ < 40) for both Cu and Zn is as high as 100%.

**Figure 4:**
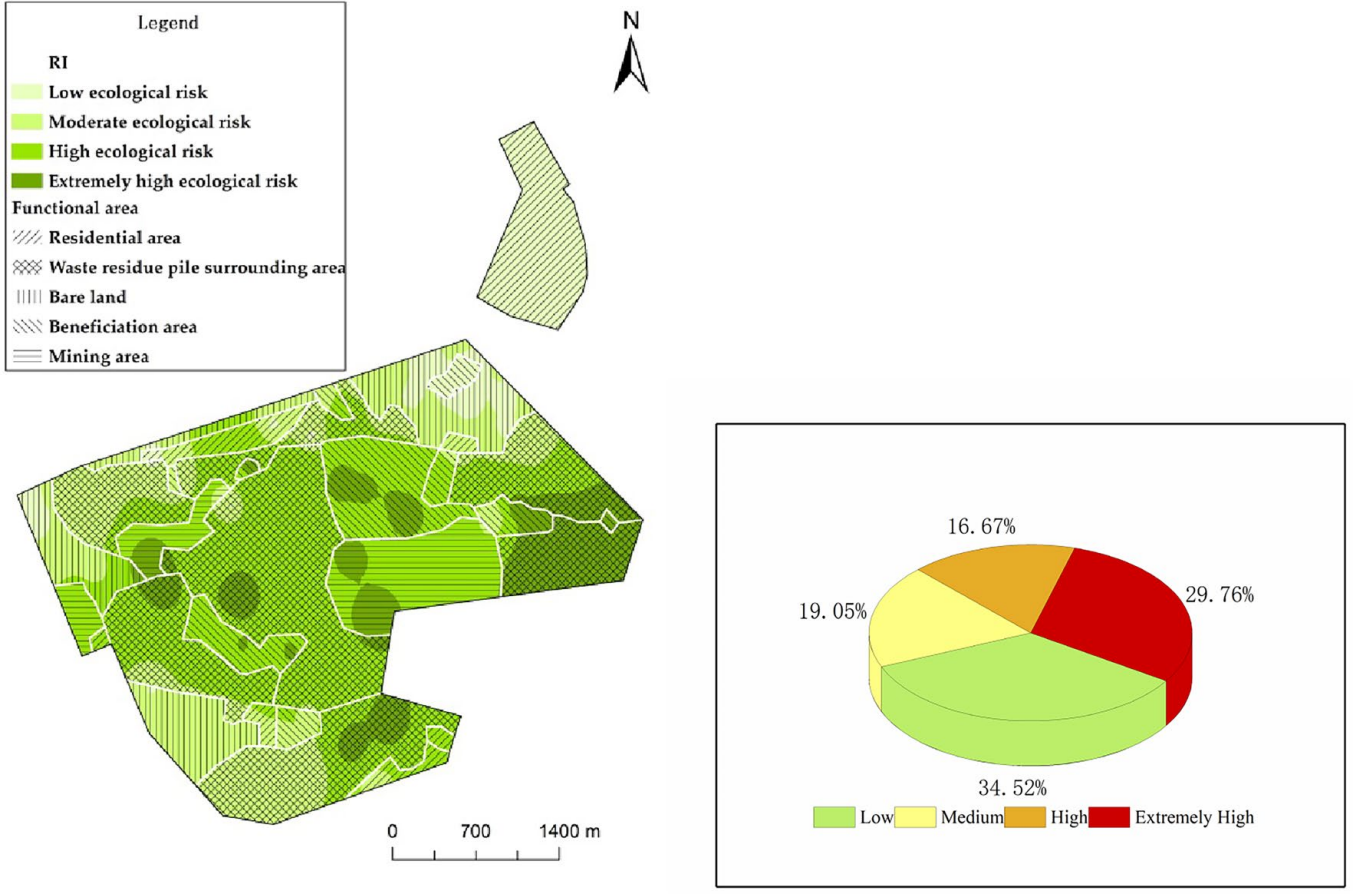
Proportion and spatial distribution of sampling points with comprehensive potential ecological risks from soil heavy metals. Map was created using ArcGIS Desktop 10.3 (https://www.esri.com/en-us/arcgis/products/arcgis-pro/overview).

The mean of the comprehensive potential ecological risk index in the study area is 269.40, reaching a high ecological risk level. The range of the risk index (RI) for all sampling points is between 41.82 and 755.01. As shown in Fig. 4, areas with low ecological risk (RI < 111) account for 34.52% of the total sampling points, followed by areas with extremely high ecological risk (RI ≥ 444), accounting for 29.76% of the total points. Areas with moderate ecological risk (111 ≤ RI < 222) and high ecological risk (222 ≤ RI < 444) account for 19.05% and 16.67% of the total points, respectively. Regions with extremely high ecological risk are mainly located in the eastern and southern parts of the study area. Among them, the areas surrounding waste piles and mining areas have a relatively concentrated distribution of sampling points with extremely high ecological risk, accounting for 36% and 48% respectively.

These results indicate that asbestos production activities in the study area have caused significant ecological risks to the soil, especially in the areas surrounding waste piles and mining areas where the ecological risk is extremely high. Among them, Ni poses a particularly strong individual potential ecological risk, with a wide range of influence and the most significant degree of harm. Gianina E. Damian et al. pointed out that an abandoned mine in Romania poses an ecological risk to the surrounding soil, with a potential ecological risk index range for Ni of 80.4 to 140.7, showing a high ecological risk^48^. The mean of the potential ecological risk for Ni (${{\text{E}}}_{{\text{r}}}^{{\text{i}}}$) obtained in this study falls within the range of Gianina E. Damian et al.’s results. Additionally, asbestos mine waste is also a major source of Ni in the surrounding soil^21^. Therefore, when assessing the suitability of agricultural land in asbestos mines and the neighboring area for crop cultivation, managers ought to direct their attention towards the Ni content of the soil.

### Probabilistic health risk of soil heavy metals

**Figure 5:**
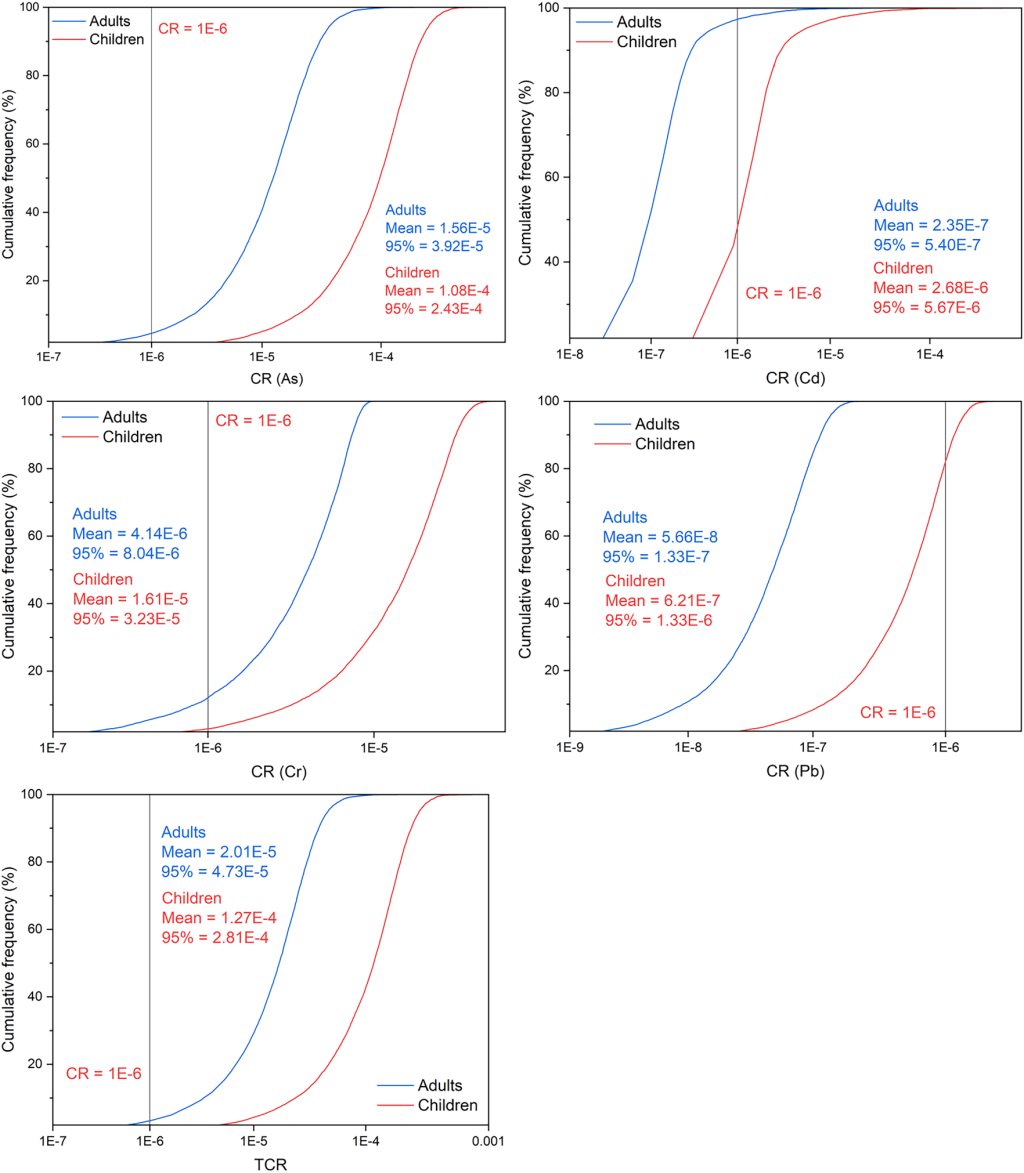
Probability distribution of carcinogenic risk from soil heavy metals.

The carcinogenic and non-carcinogenic risks for adults and children through three exposure pathways (oral ingestion, dermal contact, and inhalation) were evaluated using the health risk assessment model recommended by USEPA. As shown in Fig. 5, for adults, the average values of carcinogenic risk index (CR) for the five heavy metals are as follows: As (1.56E − 05) > Cr (4.14E − 06) > Cd (2.35E − 07) > Ni (5.74E − 08) > Pb (5.66E − 08). The mean values of CR for As and Cr are both greater than 1.00E-6, and the 95th percentile values of CR for As and Cr are 3.92E − 05 and 8.04E − 06, respectively (> N1.00E − 6). The mean values and the 95th percentile values of CR for Cd, Ni, and Pb are all less than 1.00E − 6. The maximum CR values are 3.92E − 05, 1.21E − 07, and 2.44E − 07 for Cd, Ni, and Pb, respectively. For children, the mean values of CR for the five heavy metals are as follows: As (1.08E − 04) > Cr (1.61E − 05) > Cd (2.68E − 06) > Pb (6.21E − 07) > Ni (6.27E − 08). The mean values and the 95th percentile values of CR for As, Cr, and Cd are all greater than 1.00E − 6. The mean value and 95% value of CR for Ni are 6.27E − 08 and 1.21E − 07, respectively, and the maximum CR value is 1.75E − 07. The comprehensive carcinogenic risk index (TCR) shows that the average values of TCR for adults and children are 2.00E − 5 and 1.27E − 4 (> 1.00E − 6), with 95th percentile values of TCR being 4.73E − 05 and 2.81E − 04 (> 1.00E − 6). These results indicate that there is a certain level of carcinogenic risk from heavy metals in the soil of the asbestos mine and its surrounding areas for both children and adults. Specifically, As and Cr pose carcinogenic risks to adults, while As, Cr, and Cd pose carcinogenic risks to children. The carcinogenic risk from total potentially toxic elements concentration has been reported earlier although^49^. Studies have shown that children are at higher risk of carcinogenic effects than adults in all three exposure pathways^20^. Children are more vulnerable to heavy metal contamination. In the soil of mining areas in northwestern China, the health risks of As for both children and adults exceed safety thresholds, with oral ingestion of soil particles being the main exposure pathway associated with high risk^47^. Ali Najmeddin et al. observed a carcinogenic risk of Cr for Iranian children^50^. Therefore, measures need to be taken in the polluted areas to reduce carcinogenic risks.

**Figure 6:**
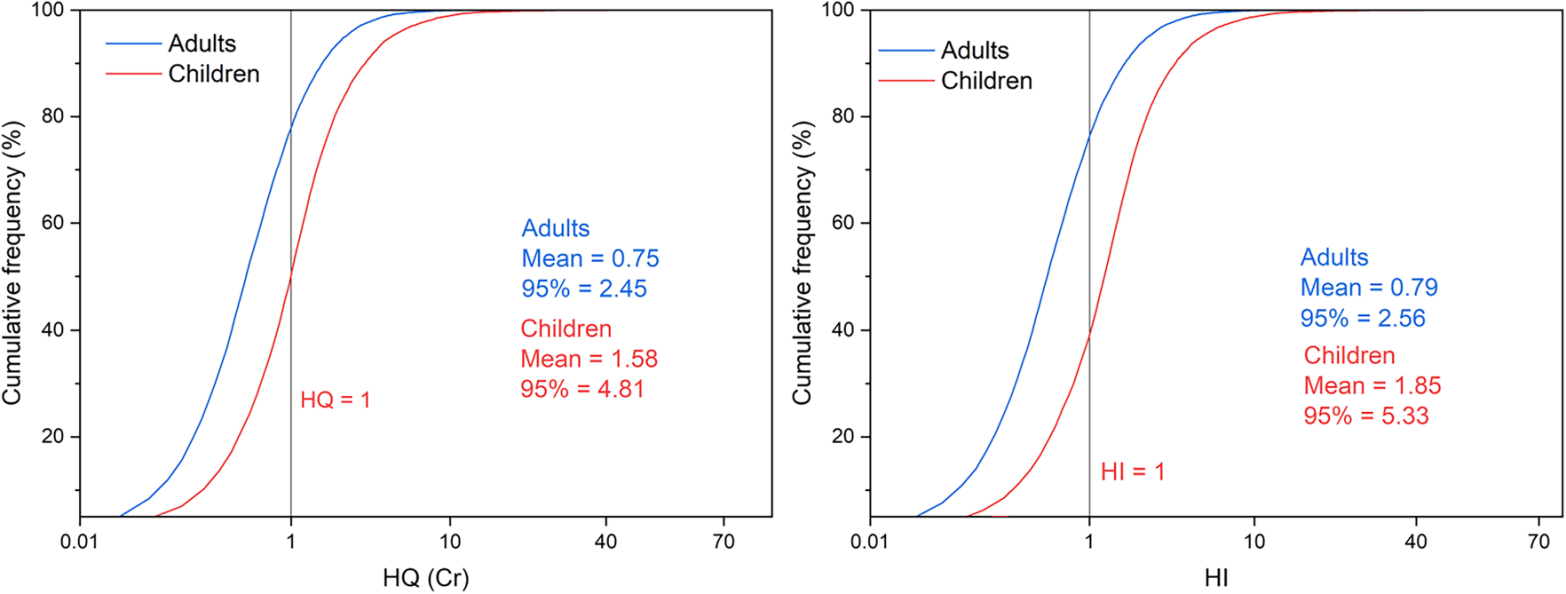
Probability distribution of non-carcinogenic risk from soil heavy metals.

As shown in Fig. 6, for adults, the mean value of non-carcinogenic risk index (HQ) for 8 heavy metals is from 4.92E − 05 to 7.51E − 01, and the 95th percentile range of HQ is from 5.84E − 05 to 2.45. For children, the mean value of non-carcinogenic risk index (HQ) for 8 heavy metals is from 1.86E − 04 to 1.58, and the 95th percentile range of HQ is from 1.83E − 04 to 4.81. In both exposure scenarios, the mean values and 95th percentile values of Cr’s HQ are greater than 1. The comprehensive non-carcinogenic risk index (HI) shows that the mean values of HI for adults and children are 7.94E − 01 and 1.85, and the 95th percentile values of HI are 2.56 and 5.33 respectively. The above results indicate that there is a non-carcinogenic risk associated with soil heavy metals in the asbestos mine and its surrounding areas for both children and adults. Specifically, Cr poses a certain degree of non-carcinogenic risk to both adults and children^51^. Some research findings also suggest that children have higher HI values than adults in various exposure pathways, revealing the fact that children are more susceptible to the harmful effects of toxic heavy metals^20,33^. Any heavy metals can be harmful even at very low concentrations, exposure time, and dosage^49^. It follows that the necessary protective measures need to be taken to reduce children's exposure and minimize the risk of exposure.

### PMF source analysis

**Figure 7:**
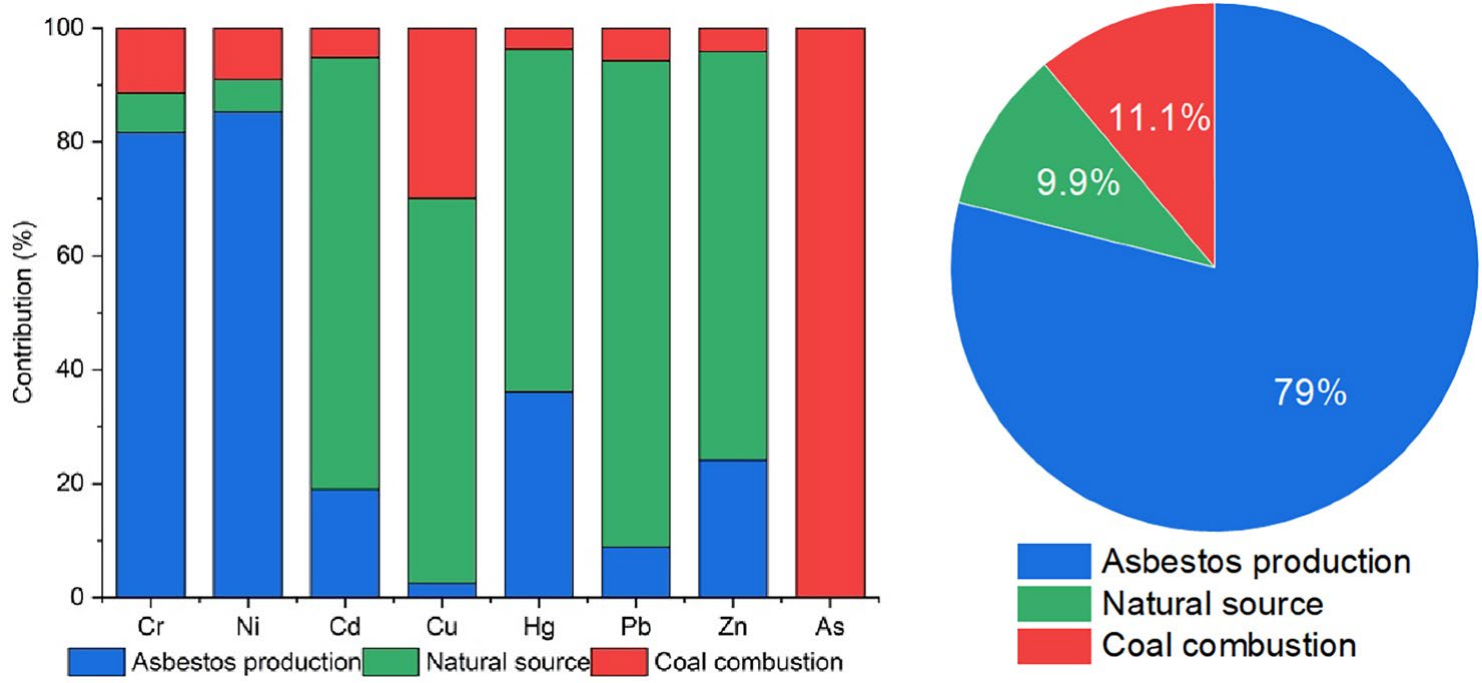
Contribution of different sources to soil heavy metals.

In this study, the PMF (Positive Matrix Factorization) source analysis model was used to calculate the spectra and contribution rates of each source component. Based on the concentration values in the source component spectra, the contribution rate of each pollution source was calculated proportionally, summarizing the contribution rates of each factor to the indicators. The results are shown in Fig. 7. The model was run 20 times under the random seed mode to determine the optimal number of factors. The simulation effects of 3–5 factors were tested separately, ensuring the minimum Q value was obtained. From the fitting results of the included model calculations, it can be seen that when the number of factors is 3, the overall concentration fit is good, with a correlation coefficient R^2^ of 0.988. In this model, the Q_robust_/Q_true_ is 0.924, indicating the robust operation of the model under stable conditions.

The data analyzed by the PMF model indicates that the pollutants with high contribution rates in Factor 1 are Cr and Ni, with contribution rates of 75.9% and 79.2%, respectively. According to Table 5, the types of asbestos pollution sources in the study area include asbestos ore, asbestos dust, asbestos tailings, and finished asbestos products. The concentration ranges of Cr and Ni are 890 ~ 1560 and 1,600 ~ 2000 mg·kg^−1^, respectively, with average concentration levels of 1339.17 and 1788.33 mg·kg^−1^, exceeding the background values of Ruoqiang County, Xinjiang^23^ by 49.3 and 67.1 times, respectively. This indicates that asbestos pollutants contain a large amount of Cr and Ni elements. It has also been reported that asbestos is often associated with heavy metal elements such as Ni and Cr^52^. Li et al. found that heavy metal Cr exhibits strong mobility, and in the soil, it shows distribution characteristics similar to asbestos minerals. Moreover, there is a severe exceedance of the standard at the asbestos tailings residue site^53^. Adarsh Kumar et al*.* found high concentrations of Cr (1148 mg·kg^−1^) and Ni (1120 mg·kg^−1^) in agricultural soils near asbestos mining waste, far exceeding the soil threshold limits^21^. These conclusions are consistent with the results of this study. Due to the production activities of asbestos mines, high concentrations of heavy metals have spread, migrated, and accumulated. Thus, Factor 1 can be considered as the asbestos pollution source, which is also consistent with the assessment results of the geoaccumulation index for Cr and Ni.

Factor 2 is characterized by the elements Hg, Cd, Pb, Cu, and Zn, with contribution rates of 59.9%, 76.2%, 86.7%, 69.0%, and 71.6% respectively. According to the detection results of asbestos pollution sources (Table 5), the average concentration levels of Hg, Cd, Pb, Cu, and Zn are 0.02, 0.07, 6.23, 10.33, and 13.33 mg·kg^−1^, respectively. These values are lower than the non-agricultural soil background values in Ruoqiang County, indicating that the concentrations of these five heavy metals in asbestos pollutants are relatively low. It has been suggested that the main sources of Cd and Cu are industrial smelting, tires, and vehicular transportation^54^, but the production activities at the study site had little effect on the concentrations of the samples, and the soil parent material factor may play a dominant role. This is consistent with the evaluation results of the geoaccumulation index (I_geo_) for Hg, Cd, Pb, Cu, and Zn. In addition, numerous studies have also indicated that Hg, Cd, Pb, Cu, and Zn mainly originate from the soil parent material. For example, in coastal soils near Shanghai^55^, sugarcane cultivation soils in northeastern Brazil^56^, agricultural soils in the Yangtze Delta region of China^57^, surface soils in regions undergoing intensive industrialization and urbanization in China^58^, and agricultural soils in a river basin in Spain^59^, similar conclusions have been drawn. Thus, Factor 2 is identified as a natural source.

Factor 3 is characterized by the element As, with a contribution rate of 100%. As is a typical indicator element of coal combustion sources^60^. After combustion, the resulting ash enters the air and settles into the soil through dry deposition. In the study area, there is a relatively large residential and industrial office area in the north, while scattered small-scale residential and office areas are distributed in the south. The prevailing wind direction is northwest, and the population consumes a large amount of coal in their daily lives. Fly ash generated from long-term coal combustion ultimately settles into the nearby soil^61^, leading to the accumulation of As in the soil in the asbestos mining environment. Furthermore, there are no other industries in the vicinity. Therefore, it can be determined that Factor 3 represents the emission source of coal combustion.

Sonali Banerjee et al.’s research indicates that natural sources (asbestos mines) and industrial sources (mining, processing, tailings) are the main contributors to Ni and Cr pollution. The contributions of transportation and agricultural emissions are 6.9% and 15.7% respectively^20^. This study identified three main sources, as shown in Fig. 7. The main contribution levels of different sources to soil heavy metals are asbestos pollution sources (79.0%) > coal combustion sources (11.1%) > natural sources (9.9%). This indicates that asbestos pollution is the major source of soil heavy metals, which is consistent with the results of Sonali Banerjee et al.’s research. The primary environmental risks at the sample sites are primarily associated with asbestos mining activities, particularly at the open pit mining site. This site produces a significant amount of dust during various operations such as blasting, shovelling, transportation, levelling, and tailings stockpiling. Consequently, it poses the highest potential risks, which should be a matter of concern. On the other hand, the potential risk triggered by soil parent material is the lowest.

## Conclusion

1. The average contents of As, Pb, Cr, Cu, and Ni in the asbestos mine and surrounding soil in the study area are 1.74, 0.13, 13.31, 0.33, and 33.37 times higher than the background values of non-agricultural land in the local area, respectively. The concentrations of Ni and Cr in the asbestos pollution source are relatively high.
2. The results of the geo-accumulation index evaluation show that there is a significant accumulation effect of Ni, Cr, and As elements, with 3.57% of the sample points being severely enriched with As. The results of the potential ecological risk index evaluation indicate that there is an extremely high comprehensive ecological risk around the waste residue piles and beneficiation area, among which Ni has an extremely strong individual potential ecological risk.
3. Soil health risk assessment shows that As and Cr pose carcinogenic risks to adults, with mean values of CR being 1.56E − 05 and 4.14E − 06, respectively. As, Cr, and Cd pose carcinogenic risks to children, with mean values of CR being 1.08E − 04, 1.61E − 05, and 2.68E − 06, respectively. Cr poses certain non-carcinogenic risks to both adults and children.
4. The PMF model identified three main sources, and their contributions to soil heavy metals are as follows: asbestos pollution source (79.0%) > coal combustion emissions source (11.1%) > natural sources (9.9%).
5. The results of this study are applicable to the risk prediction of heavy metals in soils in and around asbestos mines of the same type in China, and it is recommended that agricultural soils in and around asbestos mines should be monitored for the elements of Ni, Cr and As. In addition, it is also recommended that the management authorities should monitor and control the elements of Ni, Cr, Cd and As in soils in the vicinity of the asbestos mines, and that they should delineate the areas of restricted crowd activities and control.

## Data Availability

All data generated or analyzed during this study are included in this published article. Further information is available from the corresponding author [S.D.] upon reasonable request.
